# Clinical and Dermoscopic Factors for the Identification of Aggressive Histologic Subtypes of Basal Cell Carcinoma

**DOI:** 10.3389/fonc.2020.630458

**Published:** 2021-02-19

**Authors:** Riccardo Pampena, Gabriele Parisi, Mattia Benati, Stefania Borsari, Michela Lai, Giovanni Paolino, Anna Maria Cesinaro, Silvana Ciardo, Francesca Farnetani, Sara Bassoli, Giuseppe Argenziano, Giovanni Pellacani, Caterina Longo

**Affiliations:** ^1^ Centro Oncologico ad Alta Tecnologia Diagnostica, Azienda Unità Sanitaria Locale - IRCCS di Reggio Emilia, Reggio Emilia, Italy; ^2^ Department of Dermatology, University of Modena and Reggio Emilia, Modena, Italy; ^3^ Unit of Dermatology, IRCCS Ospedale San Raffaele, Milano, Italy; ^4^ Clinica Dermatologica, La Sapienza University of Rome, Rome, Italy; ^5^ Department of Pathological Anatomy, Modena University Hospital, Modena, Italy; ^6^ Dermatology Unit, University of Campania Luigi Vanvitelli, Naples, Italy

**Keywords:** basal cell carcinoma, subtype, infiltrative, superficial, nodular, dermoscopy

## Abstract

**Background:**

Infiltrative basal cell carcinoma (BCC) has a higher risk for post-surgical recurrence as compared to the most common low-aggressive superficial and nodular BCC. Independent diagnostic criteria for infiltrative BCC diagnosis have not been still defined. Improving the pre-surgical recognition of infiltrative BCC might significantly reduce the risk of incomplete excision and recurrence.

**Objective:**

The aim of this study is to define clinical and dermoscopic criteria that can differentiate infiltrative BCC from the most common low-aggressive superficial and nodular BCC.

**Methods:**

Clinical and dermoscopic images of infiltrative, superficial, and nodular BCC were retrospectively retrieved from our database and jointly evaluated by two experienced dermoscopists, blinded for the histologic subtype. Pairwise comparisons between the three histologic subtypes were performed and multivariable logistic regression models were constructed in order to define clinical and dermoscopic factors independently associated with each subtype. To validate our findings, two experienced dermoscopists not previously involved in the study were asked to evaluate clinical and dermoscopic images from an external dataset, guessing the proper BCC subtype between infiltrative, nodular and superficial, before and after being provided with the study results.

**Result:**

A total of 481 histopathologically proven BCCs (51.4% nodular, 33.9% superficial, and 14.8% infiltrative) were included. We found that infiltrative BCC mostly appeared on the head and neck as an amelanotic hypopigmented plaque or papule, displaying ulceration on dermoscopic examination, along with arborizing and fine superficial telangiectasia. Shiny white structures were also frequently observed. Multivariate regression analysis allowed us to define a clinical-dermoscopic profile of infiltrative BCC.

**Conclusions:**

We defined the clinical-dermoscopic profile of infiltrative BCC, allowing to differentiate this variant from superficial and nodular BCC. This will improve pre-surgical recognition of infiltrative forms, reducing the risk for post-surgical recurrence.

## Introduction

Basal cell carcinoma (BCC) is a keratinocyte carcinoma with low aggressive behavior and represents the most common tumor of human being (1). The diagnosis of BCC is generally straightforward integrating clinical and dermoscopic examination, although in a minority of cases BCC may simulate other benign and malignant tumors (2–6). Several histologic classification have been described for BCC being the superficial (sBCC), nodular (nBCC), and infiltrative (iBCC) forms the most commonly referred to. A minority of BCCs belong to a mixed pattern with more than one histotype simultaneously (7, 8). Basically, BCC histotypes can be classified as non-aggressive and aggressive depending on their behavior to deep infiltration, perineural invasion and recurrence after surgical excision (9). Among the three most common BCC histotypes, infiltrative forms are the most aggressive and it has been reported as an independent risk factor for post-surgical recurrence (10). Superficial and nodular BCCs are instead non-aggressive forms, with a very low surgical recurrence (1). Several studies described clinical and dermoscopic criteria associated to different BCC subtypes (11–15), although specific criteria allowing to differentiate the infiltrative subtype from nodular and superficial forms have not been fully elucidated (4, 6, 7, 11–13). The aim of the current study is to define clinical and dermoscopic criteria that can help to differentiate iBCC from the most common low-aggressive sBCC and nBCC.

## Materials and Methods

### Study Population

We retrospectively selected high-quality clinical and dermoscopic images of histopathologically proven BCCs from the digital databases of the Department of Dermatology of the University of Modena and Reggio Emilia (Research Project NET-2011-02347213). BCCs undergoing only partial biopsy or with more than one subtype at histopathological examination were excluded. We focused our analysis on the following histologic subtypes: infiltrative, superficial, nodular. Other subtypes only represented a minority of our case and were therefore excluded. Clinical images were taken *via* conventional clinical photography. Dermoscopic images were taken *via* polarized light contact dermoscopy (DermLite Photo 3Gen, San Juan Capistrano, CA, USA, mounted on a Canon G16 camera). Demographics and clinical data were also retrieved (i.e., skin phototype, maximum diameter and body site). This work was supported in part by Research Project NET-2011-02347213, Italian Ministry of Health. Funding source was not involved in design and conduct of the study, collection, management, analysis and interpretation of data, preparation, review, or approval of the manuscript, or decision to submit the manuscript for publication.

### Study Workflow

All clinical and dermoscopic images were jointly evaluated by two of us with different degree of expertise in dermoscopy [GaPa (novice) and RP (expert with 5 years of practice)]. Evaluators were aware of demographics and clinical data, but were blinded for the histological subtype. The following clinical parameter were evaluated: color (white, pink, red, brown, blue, black-gray) and palpability (flat, elevated, nodular) together with 12 BCC-specific dermoscopic criteria: arborizing telangiectasia, superficial fine telangiectasias, blue-gray ovoid nests, blue-gray ovoid globules, ulceration, maple leaf-like, spoke-wheel areas, concentric structures, multiple small erosion, in-focus dots, shiny red-white/structureless areas, short white streaks (chrysalis) (4). Evaluators were finally asked to classify each enrolled lesion, on clinical and dermoscopic basis, as amelanotic, light, normally or heavy pigmented according to the area covered by brown-black colors (0%, <25%, 25–75%, and >75%, respectively). To assess practical implications of our results in improving BCC histotype recognition, we selected 90 BCCs (30 iBCC, 300 nBCC, and 30 sBCC) from the database of the “Centro Oncologico ad Alta Tecnologia Diagnostica” of Reggio Emilia. Clinical and dermoscopic images of this external dataset were evaluated by two experienced Clinicians with more than 10 years training in dermoscopy (GA and GiPa) not previously involved in the study, together with demographics data. They were first blinded for study results and were asked to guess the proper histologic subtype between sBCC, nBCC, and iBCC. After a washout period of 2 weeks, they were provided with study results and repeated the same evaluation.

### Statistical Analysis

Quantitative variables were assessed for normal distribution and then compared using the Student’s T or the Mann-Whitney U test. For qualitative variables the chi-square or Fisher’s exact tests were instead used. Data were descriptively displayed and compared according to the BCC’s histologic subtype. Pairwise comparisons between the three histologic subtypes were conducted for demographics, clinical, and dermoscopic variables. Three multivariable logistic regression models were subsequently constructed, one for each pairwise comparison among histologic subtypes, to define which demographics and clinical variables and which dermoscopic features were independently associated with each of the three subtypes. Alpha level was set at 0.05, while an alpha level of 0.10 was used as cut-off for variable inclusion in multivariable models. Sensitivity, specificity, positive and negative predictive values (PPV and NPV) were calculated to define the diagnostic accuracy of the two evaluators asked to guess the proper BCC histologic subtype before and after being provided with the study results. Statistical analyses were performed using the IBM SPSS 26.0 package (Statistical Package for Social Sciences, IBM SPSS Inc., Chicago, Ill.).

## Results

**Figure 1:**
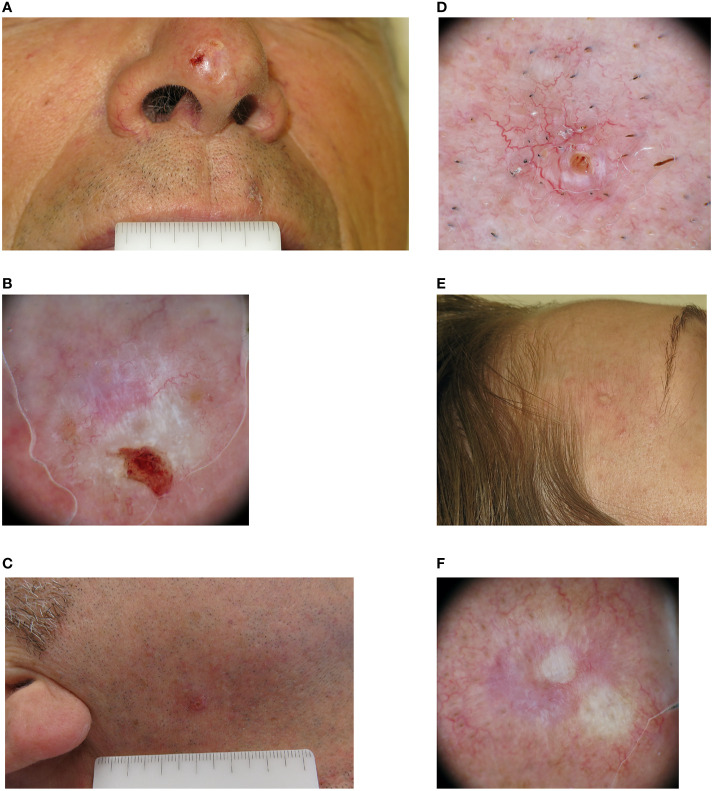
Clinical and dermoscopic images of three cases of infiltrative basal cell carcinoma. **(A)** A man in his 60s with a 7 mm amelanotic plaque located on the tip of his nose. **(B)** Dermoscopically the lesion was ulcerated, with a pinkish-whitish background. Both short white streaks and red-white structureless areas could be seen, together with superficial fine telangiectasia. **(C)** A man in his 50s with a 5 mm pinkish papule located in his right cheek. **(D)** On dermoscopic examination both classic arborizing and more superficial fine telangiectasia are seen on a pinkish background, together with a small ulceration. **(E)** A woman in her 40s with a whitish 8 mm papule located on her right temple. **(F)** Dermoscopy highlights the presence of mixed red and white structureless areas with peripheral white streaks and superficial fine telangiectasia.

A total of 526 BCCs were initially retrieved. After exclusion of 45 (8.6%) cases with mixed histotypes, 481 BCCs were enrolled belonging to 443 patients [mean age 65 years, interquartile range (IQR): 51–75 years; 218, 49.2% males and 225, 50.8% females]. Three hundred twenty-nine lesions (68.4%) belonged to patients with phototype II, 149 (31.30%) to phototype III, and 3 (0.6%) to phototype IV. Concerning histologic subtype, the majority of the enrolled BCCs were nodular (247/481; 51.4%), followed by superficial (163/481; 33.9%) and infiltrative (71/481; 14.8%) forms. Individual lesions were mainly located on the head/neck (225/481; 46.8%) and trunk (175/481; 36.4%), while only a minority arose on the limbs (upper = 41/481; 8.5%, lower = 40/481; 8.3%). Specific head and neck locations were specified in **Supplementary Table 1**. The iBCC was more frequently located on the temple and the cheek as compared to the other two histotypes. Both iBCC and the nBCC were more frequently seen on the nose than sBCC, with iBCC mainly appearing on the tip and nBCC on the nose wings. The median diameter of the enrolled lesions was 6 mm (IQR: 5–10 mm). Concerning the degree of clinical pigmentation, we found a predominance of amelanotic (198/481) and light pigmented lesions (139/481), with pink as the most widely observed color (412/481; 85.7%), followed by red (163/481; 33.9%), white (157/481; 32.6%), black-gray (122/481; 25.4%), blue (108/481; 22.5%), and brown (106/481; 22%). Dermoscopically, we found a lower number of completely amelanotic lesions (130/481; 27%), while the number of pigmented lesions proportionally increased, as compared to clinical evaluation, with a predominance of light pigmented BCCs (139/481; 28.9%). On dermoscopic examination, the most frequently observed criterion in all cases was shiny red-white structureless areas, in 339/481 (70.5%) BCCs. Multiple blue-gray globules and short white streaks were both detected in 273/481 (56.8%) lesions, arborizing telangiectasia in 264/481 (54.9%) and superficial fine telangiectasias in 144/481 (29.9%) lesions. In all, 121 (25.2%) and 112 (23.3%) out of the 481 BCCs showed blue-gray ovoid nests and maple leaf-like areas, respectively; 104/481 (21.6%) showed ulceration and 33/481 (6.9%) multiple small erosion. Other pigmented criteria, such as in focus dots, spoke-wheel areas, and concentric structures were observed only in a minority of cases. Pairwise comparisons among the three histologic subtypes are reported in **Table 1** according to demographics, clinical, and dermoscopic variables. To evaluate predictors of each BCC histologic subtype, three multivariable logistic regression models were constructed, one for each pairwise comparison. In the models of **Table 2A** demographics and clinical variables were included, together with the degree of dermoscopic pigmentation. In the models of **Table 2B** single dermoscopic criteria were instead included. We found that, as compared with sBCC, iBCC had increased odds to be elevated or nodular than flat. Clinically, iBCC also more probably occurred in older individuals, more on the head and neck region than in other body sites and more frequently displayed white and red color. Concerning dermoscopic criteria, iBCC more frequently displayed arborizing telangiectasia and ulceration than sBCCs, which was instead more characterized by superficial fine telangiectasia and multiple blue-gray globules. Comparing iBCC with nBCC, we found higher odds for nBCC to be located on the trunk and upper limbs, while iBCC more frequently appeared on the head and neck. Furthermore, nBCC more frequently appeared as a papule than iBCC. Regarding dermoscopy, superficial fine telangiectasia and ulceration were more associated with iBCC, while multiple blue-gray globules and blue-gray ovoid nests with the nBCC. Finally, we also compared superficial and nodular BCCs, showing higher odds for sBCC to be a macule and to have a larger diameter. The sBCC was also more frequently seen on the trunk and lower limbs and more frequently displayed superficial fine telangiectasia, maple-leaf areas, and multiple small erosion upon dermoscopy. The nBCC, instead, was more frequently characterized by red and black-gray color at clinical examination and by arborizing telangiectasia and blue-gray ovoid nets. The main clinical and dermoscopic differences highlighted among BCC histologic subtypes are illustrated in **Table 3** **(** **Figure 1** **).** The diagnostic accuracy of the two external readers before and after being instructed for study results is reported in **Table 4**. We registered increased levels of sensitivity and specificity and increased PPV and NPV for each of the three BCC subtypes. Baseline sensitivity for iBCC diagnosis was low for both the evaluators, with only 33.3% of cases correctly identified. After being provided with the study results almost a half of iBCC were instead correctly diagnosed.

**Table 1:** Demographics, clinical and dermoscopic variables according to the basal cell carcinoma histologic subtype with pairwise comparisons.

Variables		Histologic subtype			Total	p value superf *vs.* infiltrative	p value nodular *vs.* infiltrative	p value superf *vs.* nodular
		Infiltrative	Nodular	Superficial						
**Age**	**Median (IQR)**	71 (58–79)	67 (52–76)	61 (50–71)	**65 (51–75)**	<0.001	0.034	0.023
**Diameter**	**Median (IQR)**	7 (5–10)	6 (4–8)	6 (5–10)	**6 (5–10)**	0.267	<0.001	<0.001
**Sex**	**M**	33	128	80	**241**	0.714	0.427	0.587
		46.50%	51.80%	49.10%	**50.1%**			
	**F**	38	119	83	**240**			
	** **	53.50%	48.20%	50.90%	**49.9%**			
**Phototype**	**2**	51	167	111	**329**	0.707	0.629	0.972
		71.80%	67.60%	68.10%	**68.4%**			
	**3**	20	78	51	**149**			
		28.20%	31.60%	31.30%	**31.0%**			
	**4**	0	2	1	**3**			
	** **	0.00%	0.80%	0.60%	**0.6%**			
**Location**	**HN**	56	138	31	**225**	<0.001	<0.001	<0.001
		78.90%	55.90%	19.00%	**46.8%**			
	**Trunk**	4	79	92	**175**			
		5.60%	32.00%	56.40%	**36.4%**			
	**Upper limbs**	2	23	16	**41**			
		2.80%	9.30%	9.80%	**8.5%**			
	**Lower limbs**	9	7	24	**40**			
	** **	12.70%	2.80%	14.70%	**8.3%**			
**Palpability**	**Macule**	5	7	52	64	<0.001	<0.001	
		7.00%	2.80%	31.90%	13.31%			
	**Plaque**	55	136	108	299			
		77.50%	55.10%	66.30%	62.16%			
	**Papule**	11	104	3	118			
	** **	15.50%	42.10%	1.80%	24.53%			
**Colors clinical**	**White**	36	75	46	**157**	0.001	0.002	0.641
		50.70%	30.40%	28.20%	**32.6%**			
	**Pink**	66	200	146	**412**	0.414	0.016	0.019
		93.00%	81.00%	89.60%	**85.7%**			
	**Red**	37	98	28	**163**	<0.001	0.062	<0.001
		52.10%	39.70%	17.20%	**33.9%**			
	**Brown**	15	44	47	**106**	0.219	0.527	0.009
		21.10%	17.80%	28.80%	**22.0%**			
	**Blue**	12	62	34	**108**	0.484	0.15	0.321
		16.90%	25.10%	20.90%	**22.5%**			
	**Black-gray**	18	81	23	**122**	0.038	0.446	<0.001
	** **	25.40%	32.80%	14.10%	**25.4%**			
**Degree of clinical pigmentation**	**Non-pigmented**	36	93	69	**198**	0.134	0.056	0.016
		50.70%	37.70%	42.30%	**41.2%**			
	**Light pigmented**	17	64	58	**139**			
		23.90%	25.90%	35.60%	**28.9%**			
	**Pigmented**	12	37	16	**65**			
		16.90%	15.00%	9.80%	**13.5%**			
	**Heavy pigmented**	6	53	20	**79**			
	** **	8.50%	21.50%	12.30%	**16.4%**			
**Degree of dermatoscopic pigmentation**	**Non-pigmented**	31	55	44	**130**	0.084	0.002	0.069
		43.70%	22.30%	27.00%	**27.0%**			
	**Light pigmented**	20	80	53	**153**			
		28.20%	32.40%	32.50%	**31.8%**			
	**Pigmented**	11	42	37	**90**			
		15.50%	17.00%	22.70%	**18.7%**			
	**Heavy pigmented**	9	70	29	**108**			
	** **	12.70%	28.30%	17.80%	**22.5%**			
**Dermocopy**	**Arborizing (treelike)**	51	202	11	**264**	<0.001	0.067	<0.001
		71.80%	81.80%	6.70%	**54.9%**			
	**Short fine superficial telangiectasias**	14	8	122	**144**	<0.001	<0.001	<0.001
		19.70%	3.20%	74.80%	**29.9%**			
	**Blue-gray ovoid nests**	16	95	10	**121**	<0.001	0.013	<0.001
		22.50%	38.50%	6.10%	**25.2%**			
	**Multiple blue-gray globules**	28	141	104	**273**	0.001	0.009	0.175
		39.40%	57.10%	63.80%	**56.8%**			
	**Ulceration**	35	60	9	**104**	<0.001	<0.001	<0.001
		49.30%	24.30%	5.50%	**21.6%**			
	**Maple leaf-like**	6	46	60	**112**	<0.001	0.041	<0.001
		8.50%	18.60%	36.80%	**23.3%**			
	**Spoke-wheel areas**	1	2	18	**21**	0.013	.533*	<0.001
		1.40%	0.80%	11.00%	**4.4%**			
	**Concentric structures**	0	5	16	**21**	.004*	.591*	<0.001
		0.00%	2.00%	9.80%	**4.4%**			
	**Multiple small erosion**	1	2	30	**33**	<0.001	.533*	<0.001
		1.40%	0.80%	18.40%	**6.9%**			
	**In-focus dots**	4	14	13	**31**	0.526	>0.99*	0.357
		5.60%	5.70%	8.00%	**6.4%**			
	**Shiny red-white, structureless areas**	49	149	141	**339**	0.002	0.183	<0.001
		69.00%	60.30%	86.50%	**70.5%**			
	**Short white streaks (chrysalis)**	55	153	65	**273**	<0.001	0.015	<0.001
	** **	77.50%	61.90%	39.90%	**56.8%**			
**Total**		**71**	**247**	**163**	**481**			

IQR, interquartile range.

**Table 2:** Multivariate logistic regression analysis. Factors associated with each basal cell carcinoma histologic subtypes (infiltrative, nodular, and superficial): pairwise comparisons. Model a) demographic, clinical, and degree of pigmentation; model b) dermoscopic criteria.

A | Histotype comparison	Variables		OR	95% C.I. for OR		p value
				Lower	Upper	
Superficial *vs.* Infiltrative*	**Age**	** **	1.04	1.01	1.07	0.019
	**Location**	**HN**	ref.			<0.001
		**Trunk**	0.01	0.00	0.05	<0.001
		**Upper limbs**	0.03	0.00	0.20	<0.001
		**Lower limbs**	0.16	0.05	0.49	0.001
	**Clinical color**	**White color**	3.37	1.34	8.46	0.01
		**Red color**	7.61	2.66	21.80	<0.001
	**Surface**	**Flat**	ref.			0.007
		**Elevated**	3.77	1.12	12.77	0.033
		**Nodular**	30.05	3.48	259.36	0.002
Nodular *vs.* infiltrative**	**Location**	**HN**	ref.			<0.001
		**Trunk**	0.137	0.047	0.405	<0.001
		**Upper limbs**	0.187	0.041	0.853	0.03
		**Lower limbs**	2.197	0.715	6.748	0.169
	**Surface**	**Flat**	ref.			0.001
		**Elevated**	0.605	0.16	2.286	0.459
		**Nodular**	0.143	0.033	0.618	0.009
Superficial *vs.* nodular***	**Age**	** **	1.021	1.002	1.041	0.029
	**Diameter (mm)**		0.935	0.889	0.983	0.009
	**Location**	**HN**	ref.			<0.001
		**Trunk**	0.193	0.103	0.361	<0.001
		**Upper limbs**	0.493	0.201	1.208	0.122
		**Lower limbs**	0.078	0.024	0.254	<0.001
	**Clinical color**	**Red color**	2.587	1.318	5.077	0.006
		**Black-gray color**	3.138	1.591	6.189	0.001
	**Surface**	**Flat**	ref.			<0.001
		**Elevated**	7.107	2.827	17.866	<0.001
		**Nodular**	165.1	37.67	723.86	<0.001

B | Histotype comparison	Dermatoscopic variables	OR	95% C.I. for OR		p value
			Lower	Upper	
Superficial *vs.* infiltrative*	**Arborizing (treelike)**	17.60	5.01	61.89	<0.001
	**Superficial fine telangiectasias**	0.22	0.06	0.78	0.019
	**Multiple blue-gray globules**	0.25	0.08	0.77	0.015
	**Ulceration**	10.83	3.33	35.25	<0.001
	**Short white streaks (chrysalis)**	2.49	0.92	6.78	0.074
	**Concentric structures**	0.00	0.00	nc	0.998
	**Multiple small erosion**	0.08	0.01	0.99	0.049
Nodular *vs.* infiltrative**	**Superficial fine telangiectasias**	5.96	2.22	15.97	<0.001
	**Multiple blue-gray globules**	0.53	0.30	0.96	0.035
	**Ulceration**	3.36	1.87	6.04	<0.001
	**Blue-gray ovoid nests**	0.49	0.25	0.95	0.036
Superficial *vs.* nodular***	**Arborizing (treelike)**	15.13	6.01	38.14	<0.001
	**Superficial fine telangiectasias**	0.07	0.03	0.18	<0.001
	**Blue-gray ovoid nests**	6.61	2.33	18.74	<0.001
	**Ulceration**	3.13	0.92	10.73	0.069
	**Maple leaf-like**	0.32	0.12	0.80	0.015
	**Concentric structures**	0.20	0.04	1.05	0.057
	**Multiple small erosion**	0.04	0.00	0.62	0.021

a) *Variables entered on step 1: age. Location, white color, red color, black-gray color. Degree of dermatoscopic pigmentation. Palpability. **Variables entered on step 1: age. Location, white color, red color, pink color. Degree of dermatoscopic pigmentation. Palpability. ***Variables entered on step 1: age. Diameter (mm). Location, pink color, red color, brown color, black-gray color. Degree of clinical pigmentation. Degree of dermatoscopic pigmentation. Palpability.

b) *Variable(s) entered on step 1: arborizing (treelike) telangiectasia. Superficial fine telangiectasias. Ulceration. Maple leaf-like. Short white streaks (chrysalis). Blue-gray ovoid nests. Spoke-wheel areas. Concentric structures. Multiple small erosion. Shiny red-white structureless areas. Multiple blue-gray globules. **Variable(s) entered on step 1: arborizing (treelike) telangiectasia. Superficial fine telangiectasias. Ulceration. Maple leaf-like. Short white streaks (chrysalis). Blue-gray ovoid nests. Multiple blue-gray globules. ***Variable(s) entered on step 1: arborizing (treelike) telangiectasia. Superficial fine telangiectasias. Ulceration. Maple leaf-like. Short white streaks (chrysalis). Blue-gray ovoid nests. Spoke-wheel areas. Concentric structures. Multiple small erosion. Shiny red-white structureless areas.

**Table 3:** Infiltrative. nodular and superficial basal cell carcinoma clinical and dermoscopic profiles. Symbols (+, −, and ≈) were attributed according to the multivariate analysis results.

Variables		Infiltrative BCC *vs.*		Nodular BCC *vs.*
		Superficial	Nodular	Superficial
**Age**		+	≈	+
**Diameter**		≈	≈	−
**Location**	**HN**	++++	++*	+++
	**Trunk**	−−−−−	−−	−−
	**Upper limbs**	−−−−	−	≈
	**Lower limbs**	−−	≈	−−−
**Color (clinical)**	**White**	+	≈	≈
	**Pink**	≈	≈	≈
	**Red**	++	≈	+
	**Brown**	≈	≈	≈
	**Black-gray**	≈	≈	+
**Surface**	**Macule**	−−−−	++	−−−−−
	**Plaque**	+	≈	++
	**Papule**	++++	−−	+++++
**Dermoscopic criteria**	**Arborizing vessels**	+++	≈	+++
	**Superficial fine telangiectasia**	-	++	−−−
	**Ulceration**	++	+	+
	**Multiple blue-gray globules**	−	−	≈
	**Blue-gray ovoid nests**	≈	−	++
	**Maple leaf-like**	≈	≈	−
	**Short white streaks**	≈	≈	≈
	**Spoke-wheel areas**	≈	≈	≈
	**Concentric structures**	≈	≈	≈
	**Multiple small erosion**	≈	≈	−−−−
	**Shiny red-white structureless areas**	≈	≈	≈
	**Multiple blue-gray globules**	≈	≈	≈
**Degree of pigmentation**	**Clinical**	≈	≈	≈
	**Dermoscopic**	≈	≈	≈

*Infiltrative more on the temple. Cheek and tip of the nose; nodular more on the nose wings. Green color highlights the strongest associations, yellow is for intermediate and orange for the weakest.

**Table 4:** Diagnostic accuracy of two expert reviewers in diagnosing infiltrative. superficial and nodular basal cell carcinoma (BCC).

BCC histotype		I evaluator		II evaluator		Total	
		Before	After	Before	After	Before	After
**Infiltrative**	**Sens**	36.7%	50.0%	30.0%	46.7%	33.3%	48.3%
	**Spec**	80.0%	81.7%	76.7%	81.7%	78.3%	81.7%
	**PPV**	47.8%	57.7%	39.1%	56.0%	43.5%	56.8%
	**NPV**	71.6%	76.6%	68.7%	75.4%	70.1%	76.0%
**Superficial**	**Sens**	66.7%	70.0%	60.0%	63.3%	63.3%	66.7%
	**Spec**	80.0%	88.3%	80.0%	78.3%	80.0%	83.3%
	**PPV**	62.5%	75.0%	60.0%	59.4%	61.3%	67.2%
	**NPV**	82.8%	85.5%	80.0%	81.0%	81.4%	83.3%
**Nodular**	**Sens**	70.0%	76.7%	66.7%	73.3%	68.3%	75.0%
	**Spec**	76.7%	78.3%	71.7%	81.7%	74.2%	80.0%
	**PPV**	60.0%	63.9%	54.1%	66.7%	57.0%	65.3%
	**NPV**	83.6%	87.0%	81.1%	86.0%	82.4%	86.5%

Before and after being provided with the study results. Evaluation were performed on an external dataset of 90 BCCs (30 infiltrative, 30 nodular, and 30 superficial).

Sens, sensitivity; spec, specificity; PPV, positive predictive value; NPV, negative predictive value.

## Discussion

In this monocentric retrospective observational study, we describe the main clinical and dermoscopic features of the iBCC subtype, as compared to sBCCs and nBCCs. Clinically, we found that iBCC generally appeared as an amelanotic or hypopigmented plaque or papule, located on the head and neck, in particular on the temple, cheek, and tip of the nose. Dermoscopically, iBCC frequently displayed ulceration and a mix of arborizing and superficial fine telangiectasia. Shiny white structures were also frequently observed, such as short white streaks and red-white structureless areas. When compared with the other two histotypes, we found that patients with iBCC were slightly older than those with sBCC, but no age differences were observed with nBCC. Also, the iBCC was more often located on the head and neck and significantly less on the trunk and upper limbs, compared to the other non-aggressive histotypes. Concerning the degree of pigmentation seen on dermoscopy, iBCC was significantly more amelanotic and less heavy pigmented than nBCC in univariate analysis. However, when controlling for age, location, palpability, and clinical color in multivariate analysis no significant differences were observed. As expected, iBCC was more frequently palpable (plaque or papule) than the sBCC and less than nBCC.

Regarding dermoscopic examination, we found a prevalence of arborizing telangiectasia in iBCC, as compared to sBCC, in which superficial fine telangiectasia were instead more frequently seen. No significant differences in arborizing telangiectasia were instead observed between iBCC and nBCC, while in the former superficial fine telangiectasia were more frequently observed. Ulceration was more often reported in iBCC than both sBCC and nBCC, while multiple blue-gray globules and blue-gray ovoid nests were rarely seen among iBCCs. The definition of a specific Clinicians are dermoscopic profile for iBCC, sBCC, and nBCC, allowed external readers to increase their diagnostic accuracy in differentiating these histotypes after being provided with our study results. In particular, they were able to correctly identify a higher number of iBCCs (increased sensitivity). with a reduction of iBCCs misdiagnosed as sBCCs or nBCCs (false negative cases).

In clinical practice, this would improve pre-surgical recognition of iBCC, allowing the surgeon to keep wider margins and reducing the risk of recurrence. Previous studies mainly defined clinical, demographic and dermoscopic features associated with sBCC (11–13). However, little is known about factors allowing to differentiate sBCC from iBCC. The sBCC has been shown to occur in younger patients than the other BCC histotypes and to be mainly located in non-chronically sun-exposed areas, such as the trunk (16). Concerning dermoscopy, multiple small erosions, superficial fine telangiectasia and structures corresponding to dermo-epidermal pigmentation were shown to predict sBCC subtype. However, the presence of blue-gray ovoid nests seems to exclude the diagnosis of sBCC (12). Dermoscopic criteria more associated with iBCC have been previously reported. However, these findings are mainly based on descriptive analysis and expert opinions, while independent clinical and dermoscopic predictors have not been defined by multivariable analysis so far (4, 6, 11–13, 17). In 2014, Longo and colleagues reported on a study population of 22 iBCCs, 22 nBCC and 44 sBCC, that infiltrative forms were featured by arborizing telangiectasia, superficial fine telangiectasia and shiny white-red structureless areas (11). However, none of these criteria was significantly more observed in iBCC as compared to the other histotypes because of the small number of cases analyzed. Furthermore, multivariable logistic regression analysis was only performed to define confocal criteria predictive of each histotype.

Our study fills this gap by focusing on clinical and dermoscopic criteria independently associated with sBCC, nBCC and iBCC subtypes. In 2020, Conforti and colleagues defined the dermoscopic criteria independently associated with the sclerodermiform BCC subtype as compared to the other subtypes (sBCC + nBCC). They found in multivariate analysis, that ulceration was significantly more frequently seen in sclerodermiform BCC, followed by fine arborizing telangiectasia, pink-white areas and multiple blue-gray dots and globules (14). Recently, a systematic review pointed out that no very specific dermoscopic criteria allow to differentiate different BCC histotypes (7). The authors reported that nBCC was more characterized by arborizing telangiectasia (75%), shiny white structures (43%), and ulceration (31%), while iBCC mainly presented arborizing telangiectasia (76%), ulceration (44%), and short-fine telangiectasia (40%). Only two dermoscopic structures appeared to be relatively unique for one subtype: leaf-like areas and shiny white-red structureless background in sBCC. In our study we failed to find these two criteria as more associated with sBCC, however, we confirmed that sBCC is easier to differentiate from both nBCC and iBCC. Wider differences were indeed observed in multivariable analysis in term of anatomic location, palpability and dermoscopic criteria, when comparing sBCC with nBCC and iBCC. Furthermore, we also reported significant differences between nBCC and iBCC. In particular iBCC was more frequently located on the head and neck as a macule, while nBCC was more frequently seen on the trunk as a papule. Upon dermoscopy, the most important difference regarded the highest occurrence of superficial fine telangiectasia in iBCC. This confirms previous observations, describing the telangiectasia of iBCC as having smaller caliber and less tendency to branch than those of nBCC (6). However, we didn’t find significant differences in classic arborizing telangiectasia between iBCC and nBCC. Thus, we can conclude that in iBCC superficial fine and arborizing telangiectasia often coexist in the same lesion.

Some limitations of the current study include the retrospective design, the exclusion of minor BCC histotypes and lack of histopathological specimens’ re-assessment. The latter limitation could have influenced the histotype recognition as well as the proportion of lesions showing more than one histotype. We partially controlled for this limitation by asking the pathologist (AMC) for re-assessment in case of doubtful lesions. Another limitation of the current study is the over-representation of patients with photo-type II or III, which is due to the phenotypic characteristics of the Italian population.

To conclude, we defined a clinical-dermoscopic profile of iBCC, allowing to differentiate this variant from sBCC and nBCC when Clinicians are trained on the results of the dermoscopic findings of our study.

## Data Availability Statement

The raw data supporting the conclusions of this article will be made available by the authors, without undue reservation.

## Ethics Statement

The study involving human participants was reviewed and approved by Comitato Etico dell’Area Vasta Emilia Nord—Modena, Italy. Protocol number NET‐2011‐02347213. The patients/participants provided their written informed consent to participate in this study.

## Author Contributions

CL, RP, GaPa equally contributed to the study concept and design, data analysis and interpretation, and writing of the report. RP did the statistical analysis. AC did the histopathological reassessment of doubtful cases. SBo, ML, GiPa, AC, SC, FF, SBa, GA, GiPe contributed to the data interpretation and provided expert insight into the writing of the report. All authors contributed to the article and approved the submitted version.

## Funding

This work was supported in part by Research Project NET-2011-02347213, Italian Ministry of Health. Funding source was not involved in the design and conduct of the study, collection, management, analysis and interpretation of data, preparation, review, or approval of the manuscript, or decision to submit the manuscript for publication.

## Conflict of Interest

The authors declare that the research was conducted in the absence of any commercial or financial relationships that could be construed as a potential conflict of interest.
