# Supplementary material for: Clinical and Dermoscopic Factors for the Identification of Aggressive Histologic Subtypes of Basal Cell Carcinoma
Source: Front Oncol. 2021 Feb 19;10:630458. doi: 10.3389/fonc.2020.630458 (PMC7933517; doi:10.3389/fonc.2020.630458)
Supplement: Supplementary file 1 [file Table_1.docx]

Supplementary Material

**Supplementary Table 1:** Specific head and neck location according to the basal cell carcinoma histologic subtype.

| **Body site** | | | | **Histologic subtypes** | | | **Total** |
| --- | --- | --- | --- | --- | --- | --- | --- |
|  |  |  |  | **Infiltrative** | **Nodular** | **Superficial** |  |
| **Head and neck** | | | | **56** | **138** | **31** | **225** |
|  |  |  |  | **76.0%** | **55.5%** | **19.0%** | **46.8%** |
| **Neck** | | | | **3** | **5** | **1** | **9** |
|  |  |  |  | **4.20%** | **2.00%** | **0.60%** | **1.90%** |
| **Scalp** | | | | **4** | **17** | **5** | **26** |
|  |  |  |  | **5.6%** | **6.9%** | **3.1%** | **5.4%** |
| **Face** | **Total** | | | **49** | **116** | **25** | **190** |
|  |  |  |  | **66.2%** | **46.6%** | **15.3%** | **39.5%** |
|  | **Upper face** | **Total** | | **17** | **33** | **10** | **60** |
|  |  |  |  | **24.0%** | **13.3%** | **6.2%** | **12.5%** |
|  |  | **forehead** | | 6 | 23 | 5 | 34 |
|  |  |  |  | 8.50% | 9.30% | 3.10% | 7.10% |
|  |  | **temple*** | | 11 | 10 | 5 | 26 |
|  |  |  |  | 15.50% | 4.00% | 3.10% | 5.40% |
|  | **Central face** | **Total** | | **29** | **75** | **14** | **118** |
|  |  |  |  | **38.0%** | **30.1%** | **8.5%** | **24.60%** |
|  |  | **cheek** | | 8 | 8 | 5 | 21 |
|  |  |  |  | 11.30% | 3.20% | 3.10% | 4.40% |
|  |  | **cheekbone*** | | 0 | 3 | 0 | 3 |
|  |  |  |  | 0.00% | 1.20% | 0.00% | 0.60% |
|  |  | **pre-auricular*** | | 2 | 4 | 1 | 7 |
|  |  |  |  | 2.80% | 1.60% | 0.60% | 1.50% |
|  |  | **ear*** | | 2 | 3 | 1 | 6 |
|  |  |  |  | 2.80% | 1.20% | 0.60% | 1.20% |
|  |  | **peri-ocular*** | | 3 | 11 | 1 | 15 |
|  |  |  |  | 4.20% | 4.40% | 0.60% | 3.20% |
|  |  | **nose*** | ***Total*** | ***14*** | ***46*** | ***6*** | ***66*** |
|  |  |  |  | ***19.7%*** | ***18.5%*** | ***3.6%*** | ***13.7%*** |
|  |  |  | ***Base*** | *0* | *3* | *0* | *3* |
|  |  |  |  | *0.00%* | *1.20%* | *0.00%* | *0.60%* |
|  |  |  | ***Piramid*** | *0* | *3* | *0* | *3* |
|  |  |  |  | *0.00%* | *1.20%* | *0.00%* | *0.60%* |
|  |  |  | ***Back*** | *3* | *8* | *1* | *12* |
|  |  |  |  | *4.20%* | *3.20%* | *0.60%* | *2.50%* |
|  |  |  | ***Tip*** | *6* | *10* | *1* | *17* |
|  |  |  |  | *8.50%* | *4.00%* | *0.60%* | *3.50%* |
|  |  |  | ***Wing*** | *2* | *16* | *3* | *21* |
|  |  |  |  | *2.80%* | *6.50%* | *1.80%* | *4.40%* |
|  |  |  | ***Nasal fold*** | *3* | *6* | *1* | *10* |
|  |  |  |  | *4.20%* | *2.40%* | *0.60%* | *2.10%* |
|  | **Lower face** | **Total** | | **3** | **8** | **1** | **12** |
|  |  |  |  | **4.2%** | **3.2%** | **0.6%** | **2.4%** |
|  |  | **nasal-labial*** | | 1 | 1 | 0 | 2 |
|  |  |  |  | 1.40% | 0.40% | 0.00% | 0.40% |
|  |  | **lip*** | | 0 | 3 | 0 | 3 |
|  |  |  |  | 0.00% | 1.20% | 0.00% | 0.60% |
|  |  | **jaw*** | | 1 | 3 | 0 | 4 |
|  |  |  |  | 1.40% | 1.20% | 0.00% | 0.80% |
|  |  | **chin*** | | 1 | 1 | 1 | 3 |
|  |  |  |  | 1.40% | 0.40% | 0.60% | 0.60% |
| **Trunk** | | | | **4** | **79** | **92** | **175** |
|  |  |  |  | **5.60%** | **32.00%** | **56.40%** | **36.40%** |
| **Limbs** | | | | **11** | **30** | **40** | **81** |
|  |  |  |  | **15.5%** | **12.1%** | **24.5%** | **16.8%** |
| **Total** | | | | **71** | **247** | **163** | **481** |

*High-risk areas (H area).
